# Supplementary material for: Positive In Vitro Effect of ROCK Pathway Inhibitor Y-27632 on Qualitative Characteristics of Goat Sperm Stored at Low Temperatures
Source: Animals (Basel). 2024 May 12;14(10):1441. doi: 10.3390/ani14101441 (PMC11117216; doi:10.3390/ani14101441)
Supplement: Supplementary file 1 [file animals-14-01441-s001.zip › animals-3002700-supplementary.pdf]

Table S1. Differential metabolites of goat sperm between Y-27632 group and control group.

Name	RT	MZ	VIP	*P*-value	FC
L-Lactic acid	133.891	89.0246455	1.59765039	0.01324752	0.79613995
cis,cis-Muconic acid	202.124	141.017654	1.50108415	0.02231381	0.89186374
Itaconic acid	254.951	129.019709	1.49954683	0.02567359	0.88448404
Allopurinol-1-ribonucleoside	129.574	269.086657	1.89097145	0.00640433	0.89395234
Gallic acid	23.1374	169.018331	1.60305019	0.01623787	0.81468869
SM(d18:1/16:0)	114.263	703.571584	1.73186466	0.02052285	0.80129146
L-Glutamic acid	233.331	146.046461	1.27994411	0.04672727	0.80082779
L-Malic acid	237.155	133.014766	2.18593308	0.00076379	0.68312241
L-Palmitoylcarnitine	103.043	400.339049	1.9317731	0.00209023	0.85964016
Propionylcarnitine	171.971	218.13747	1.70235611	0.00533164	1.14907337
Indolelactic acid	62.9337	204.067583	2.22982898	0.00008847	0.68805644
Gingerol	15.178	293.180347	1.40672123	0.02641954	0.66844547
Salviaflaside methyl ester	130.129	537.164086	1.75910088	0.01211489	0.83888769
Hydroxyphenyllactic acid	106.166	181.051268	2.17860415	0.00011832	0.72728677
PC(22:5(4Z,7Z,10Z,13Z,16Z)/18:0)	39.5299	836.607492	1.53736094	0.02624390	0.86279357
2',4'-Dihydroxyacetophenone	117.241	151.040632	2.36023093	0.00000267	1.70691396
Erythrulose	17.8604	165.041564	1.49379247	0.04813945	0.66951219
PC(22:5(7Z,10Z,13Z,16Z,19Z)/18:1(11Z))	21.155	834.592336	1.57245355	0.01550070	0.8063452
Succinic anhydride	222.478	99.009182	1.73405992	0.01866335	0.88760327
trans-Aconitic acid	254.869	173.009548	1.43978879	0.03907058	0.88249207
Pyrrolidine	182.913	72.0803042	1.37147252	0.03216485	0.8602252
Glycerol	49.0764	91.0403299	1.49602052	0.02760388	0.82478496
4-Hydroxyphenylpyruvate	40.439	179.035394	1.70894906	0.00861398	0.82490118
Decanoylcarnitine	119.621	316.246038	1.62625	0.00232910	1.70701392
Hydroxypropionic acid	35.6959	89.0246728	1.94478092	0.00159602	0.84115341
2-Methylbenzoic acid	54.1835	135.045578	2.54435808	0.00000077	2.39718838
Phenyllactic acid	41.6306	165.05619	2.12899048	0.00030842	0.73738444
Threonic acid	188.653	135.030305	1.38826469	0.03650358	0.78095966
PC(22:4(7Z,10Z,13Z,16Z)/16:0)	21.49945	810.595661	1.70876997	0.00655316	0.82493022
SM(d18:1/24:1(15Z))	128.261	813.679446	1.75493038	0.01231242	0.74011976
Butyrylcarnitine	159.834	232.152939	2.22239953	0.00000875	1.26848504
Dihydroxyacetone phosphate	250.769	168.991321	1.99829326	0.00199501	0.82436614
Cholesterol sulfate	15.1798	465.304792	1.40636932	0.04381911	0.81215428
PC(P-18:1(9Z)/15:0)	110.789	730.591612	1.62718169	0.01607234	0.77854557
Isocrotonic acid	167.267	87.0434678	1.4443396	0.03856951	0.85206082
PE(16:0/18:2(9Z,12Z))	48.821	714.508096	1.93606174	0.00784278	0.70973609
Betaine aldehyde	157.264	102.090595	1.59682027	0.00961540	0.78903669
SM(d16:1/24:1(15Z))	111.552	785.647166	1.61443194	0.03932800	0.74390733
SM(d18:0/18:0)	112.96	733.609821	1.81176267	0.01415814	0.75413691
gamma-Aminobutyric acid	233.306	104.06987	1.34699788	0.04153755	0.90077592
L-Glutamine	250.243	147.075463	1.42852017	0.04038471	0.95866416

Leucinic acid	49.5423	131.071894	1.51031485	0.01441341	0.74806815
D-Glutamine	220.568	145.062519	1.41755549	0.02910294	0.82792313
Taurine	177.641	124.007856	1.28721403	0.04656506	0.83607269
Trehalose	216.208	341.110559	1.58664716	0.02402891	0.69099304
SM(d18:0/18:1(9Z))	154.054	731.603228	1.79944416	0.03646287	0.46259078
PC(22:6(4Z,7Z,10Z,13Z,16Z,19Z)/18:1(11Z))	20.438	832.578686	1.81098055	0.00734346	0.72189456
1,2,3-Trihydroxybenzene	153.538	125.0249	1.50930472	0.01765315	0.80317989
D-Xylose	176.306	149.045993	1.60922234	0.04297202	0.72145179
Bergapten	153.457	215.033764	1.57067647	0.03341977	0.95426107
Byssochlamic acid	41.096	331.120379	2.11829732	0.00156854	0.54843215
PC(22:4(7Z,10Z,13Z,16Z)/P-18:1(11Z))	38.1118	820.612789	1.52761503	0.03808034	0.8733693
3-Hydroxycinnamic acid	106.256	163.040634	2.12165556	0.00023919	0.7419781
PC(P-18:1(11Z)/20:5(5Z,8Z,11Z,14Z,17Z))	20.878	790.566099	1.64965152	0.02324982	0.84859034
SM(d18:1/14:0)	115.565	675.539984	1.92321024	0.00796248	0.84070823
Hydroxyprolyl-Hydroxyproline	242.542	245.111317	1.38277432	0.02915285	0.79266905
N-Palmitoylsphingosine	18.4286	538.515108	1.83454965	0.00185802	0.74895449
L-Nicotine	34.8885	163.121899	1.95073356	0.00250932	3.11100302
N-(5-Methyl-3-oxohexyl)alanine	219.434	202.142624	1.67667643	0.01208735	0.88445697
PC(P-18:0/16:1(9Z))	40.01325	744.583314	1.60396629	0.04755624	0.66663773
L-Iditol	91.5338	181.072439	1.57888642	0.01665826	0.82534491
Bergenin	171.586	373.074547	1.54305603	0.01872220	1.30984682
4-Imidazolone-5-propionic acid	203.7765	157.059593	1.90256706	0.00135156	0.74394448
Pyruvic acid	73.9164	87.0091344	1.73602835	0.00543077	0.91758718
N-(2-Furoyl)glycine	233.683	168.028313	1.69766577	0.01602441	0.94450477
(6beta,22E)-6-Hydroxystigmasta-4,22-dien-3-one	98.7348	427.357736	1.90181881	0.00181325	0.81037178
Epidermin	235.382	262.126563	1.5819098	0.01569775	1.17625927
4-Trimethylammoniobutanoic acid	223.063	146.116763	1.61972072	0.01331600	0.8758921
Isocitric acid	266.349	173.009806	1.4456357	0.03209878	0.87534763
Betaine	165.3	118.085529	1.44558995	0.03441459	0.92036645
Oxidized-adrenal-ferredoxin	210.215	184.092884	2.07733833	0.00021467	0.67849278
(1S,2S,4R,8R)-p-Menthane-1,2,9-triol	26.8294	189.147003	1.58260697	0.01279514	1.44502675
Deoxyribose 5-phosphate	133.896	213.022233	1.49877015	0.02472029	0.83690911
L-Octanoylcarnitine	128.894	288.214717	2.46286458	0.00000007	1.78957089
Naringenin	133.895	307.036187	1.73840051	0.00519099	0.86835192
Aesculin	42.40785	339.072766	1.62708136	0.00722120	0.66453606
5-Aminopentanal	210.219	102.090557	2.28468398	0.00010537	0.79522113
PE(20:2(11Z,14Z)/14:0)	48.6721	716.515319	1.63754648	0.02739182	0.86692028
Panaquinquecol 4	207.417	275.158686	1.39390153	0.04006072	0.8783675
o-Cresol	40.4397	107.05052	1.78090464	0.00557429	0.8207636
Calystegine B2	144.17	176.090564	1.7367159	0.00586044	0.73273736
9,10-Epoxyoctadecenoic acid	27.79005	295.228954	2.16552698	0.00003427	0.50883506
cis-5-Tetradecenoylcarnitine	107.575	370.292822	1.97983075	0.00119355	0.81352983
Tryptophyl-Proline	155.2235	302.147927	1.68495379	0.00781944	0.76845153

PE(18:0/14:1(9Z))	51.609	690.50073	2.00177361	0.00308607	0.78015166
7,8-Dehydro-beta-micropteroxanthin	104.92	395.296064	1.64292118	0.01000877	0.61435799
PC(16:0/15:0)	50.6692	720.538132	1.65899162	0.03028136	0.8174672
2-Oxo-4-methylthiobutanoic acid	31.1015	147.012488	1.482562	0.02889540	0.83078657
Koeniginequinone A	238.567	242.077754	1.53775375	0.02568826	0.93271057
Phenylalanyl-Methionine	106.893	297.127274	1.864503	0.00247919	1.48644057
Isocaproic acid	24.71285	117.090103	2.10472657	0.00140485	1.64053914
25-Hydroxycholesterol	101.235	425.342195	1.9169239	0.00127904	0.79242571
Oxymorphone	245.491	302.132378	2.20629148	0.00015070	0.76431171
LysoPE(0:0/22:5(4Z,7Z,10Z,13Z,16Z))	116.2625	528.305063	1.86205401	0.00066887	0.4990716
Bovinic acid	49.4644	279.233432	1.85820738	0.00347730	0.67627413
Tryptophyl-Valine	261.0695	304.159419	1.54700766	0.01577616	0.82981681
Calcitriol	103.468	399.326585	2.03730669	0.00069627	0.80819253
PE(22:1(13Z)/14:1(9Z))	46.6432	744.546184	1.76965912	0.00770739	0.79043918
Squamolone	90.29785	129.065047	1.32205734	0.03813924	1.38284009
3-Methyl-2-oxopentanoate	28.4272	129.056205	2.10109174	0.00044151	0.65023568
3-Hydroxyisovalerylcarnitine	186.85	262.163634	1.8321443	0.00493021	0.8583339
Methylmalonic acid semialdehyde	210.219	103.038249	2.36848323	0.00000950	0.81881615
(4-Hydroxybenzoyl)choline	303.6305	224.126349	1.34595446	0.04363992	0.73128929
SM(d18:1/18:1(9Z))	110.877	729.583891	1.42866717	0.03567001	0.81739508
Gynocardin	197.988	304.098875	1.31673875	0.04966026	1.19275418
LysoPE(0:0/18:0)	125.55	482.322074	1.56724826	0.02063716	0.83897397
trans-Hexadec-2-enoyl carnitine	103.5	398.324472	2.05876836	0.00057043	0.80981743
PI(20:3(5Z,8Z,11Z)/18:1(11Z))	124.235	887.559422	1.49772861	0.02847439	0.79573375
9,10-epoxyoctadecanoic acid	27.7866	297.244059	2.15929556	0.00002729	0.59758447
Acetylglycine	26.47365	116.035813	1.25595605	0.04826434	0.83531139
Myristic acid	26.1749	227.20273	2.27696484	0.00000886	0.69786657
Stearic acid	20.4784	283.265408	1.77110876	0.02665119	0.73508209
LysoPC(20:0/0:0)	118.942	552.399076	1.41839302	0.04012743	0.83440764
beta-Alanine	165.5535	88.0407902	1.27948721	0.04868070	0.80041283
(25S)-26-Hydroxy-24-methylenecycloartan-3-one	94.69145	455.390393	2.28558572	0.00000004	2.17329446
2-Acetylpyrazine	31.001	123.054497	2.44681402	0.00000001	2.33687186
4-(4-Hydroxyphenyl)-2-butanone glucoside	130.129	327.139479	1.67650415	0.02443232	0.91996558
Citrulline	218.038	176.101725	1.72505389	0.00692877	0.76280652
1-Isothiocyanato-7-(methylsulfinyl)heptane	194.03	220.080109	1.19820421	0.03086210	0.24818323
4-Hydroxybenzyl isothiocyanate rhamnoside	196.325	312.090325	1.65247746	0.00754396	0.72948656
trans-Cinnamic acid	41.6919	147.045635	2.06385891	0.00063168	0.72579964
Docosa-4,7,10,13,16-pentaenoyl carnitine	91.1821	474.355532	1.93538332	0.00161277	0.77803099
4-(Methylthio)-1-butanol	215.9015	121.078371	1.33269236	0.03493340	1.26321993
2,5-Dihydro-2,4-dimethyloxazole	27.9266	100.074942	1.77894668	0.00376251	1.44160054
2,3,4,5-Tetrahydropiperidine-2-carboxylate	31.5205	128.082904	2.46452875	0.00000006	3.38071153
3-Hydroxy-beta-ionone	18.332	209.15195	1.87244761	0.00443354	1.34500236
Palmitoleic acid	49.3616	253.218309	2.13267487	0.00014764	0.54884374

2-Amino-4-oxo-4-alpha-hydroxy-6-(erythro-1',2',3'-trihydroxypropyl)-5,6,7,8-tetrahydroxypterin	149.827	322.09633	1.34014718	0.02560379	1.20776678
a-Amino-2,5-dihydro-5-oxo-4-isoxazolepropanoic acid N2-glucoside	179.855	335.103417	1.28893114	0.04416264	0.84020253
LysoPE(20:4(8Z,11Z,14Z,17Z)/0:0)	117.624	502.28869	2.32682311	0.00000366	0.37678891
L-Hexanoylcarnitine	141.314	260.183956	2.45877692	0.00000001	1.87635521

RT, the chromatographic retention time of the substance; MZ, the mass to charge ratio of characteristic ions in a substance; VIP, variable importance in projection; *P*-value, obtained from the t-test of the substance in this group comparison; FC, fold change.

Table S2. Enrichment analysis of differential metabolites in goat sperm from Y-27632 group and control group.

Name	*P*-value	Number	Name	*P*-value	Number
Metabolic pathways	0.0005717	39	Purine metabolism	0.6320106	1
ABC transporters	0.0000491	8	Valine, leucine and isoleucine biosynthesis	0.2056172	1
Carbon metabolism	0.0008045	6	Tyrosine metabolism	0.5441961	1
Biosynthesis of amino acids	0.0014754	6	Phosphonate and phosphinate metabolism	0.4302910	1
Central carbon metabolism in cancer	0.0000012	6	Glutathione metabolism	0.3168086	1
D-Amino acid metabolism	0.0004664	5	Starch and sucrose metabolism	0.3098947	1
Glyoxylate and dicarboxylate metabolism	0.0003242	5	Amino sugar and nucleotide sugar metabolism	0.6970690	1
Biosynthesis of cofactors	0.2235488	5	Neomycin, kanamycin and gentamicin biosynthesis	0.5578916	1
Pentose and glucuronate interconversions	0.0024267	4	Inositol phosphate metabolism	0.3760650	1
Glycerophospholipid metabolism	0.0021312	4	Glycosylphosphatidylinositol (GPI)-anchor biosynthesis	0.0391573	1
Propanoate metabolism	0.0005950	4	Arachidonic acid metabolism	0.5300865	1
2-Oxocarboxylic acid metabolism	0.0426780	4	alpha-Linolenic acid metabolism	0.3568934	1
Retrograde endocannabinoid signaling	0.0000294	4	Sphingolipid metabolism	0.2214053	1
Glucagon signaling pathway	0.0001077	4	Thiamine metabolism	0.2669531	1
Glycolysis / Gluconeogenesis	0.0033813	3	Vitamin B6 metabolism	0.2520675	1
Citrate cycle (TCA cycle)	0.0009253	3	Porphyrin metabolism	0.7775908	1
Fatty acid biosynthesis	0.0192801	3	Terpenoid backbone biosynthesis	0.3697373	1
Arginine biosynthesis	0.0014079	3	Sulfur metabolism	0.2815492	1
Pyrimidine metabolism	0.0249876	3	Biosynthesis of unsaturated fatty acids	0.5252892	1

Alanine, aspartate and glutamate metabolism	0.0025159	3	Fatty acid metabolism	0.7092584	1
Glycine, serine and threonine metabolism	0.0115738	3	cAMP signaling pathway	0.2214053	1
Taurine and hypotaurine metabolism	0.0014079	3	FoxO signaling pathway	0.0487102	1
Linoleic acid metabolism	0.0025159	3	Sphingolipid signaling pathway	0.1392782	1
Pyruvate metabolism	0.0037054	3	Phospholipase D signaling pathway	0.1041095	1
Neuroactive ligand-receptor interaction	0.0143882	3	Autophagy - other	0.0295108	1
Proximal tubule bicarbonate reclamation	0.0005635	3	Autophagy - animal	0.0581703	1
Protein digestion and absorption	0.0109252	3	AMPK signaling pathway	0.1976063	1
Pentose phosphate pathway	0.0466306	2	Ferroptosis	0.2520675	1
Fructose and mannose metabolism	0.0997576	2	Necroptosis	0.0951003	1
Galactose metabolism	0.0757831	2	Gap junction	0.1041095	1
Ascorbate and aldarate metabolism	0.1092381	2	Circadian entrainment	0.0860026	1
Primary bile acid biosynthesis	0.0786657	2	Thermogenesis	0.2056172	1
Cysteine and methionine metabolism	0.1389882	2	Long-term potentiation	0.0675385	1
Lysine degradation	0.0875165	2	Synaptic vesicle cycle	0.1130310	1
Arginine and proline metabolism	0.1597131	2	Long-term depression	0.0860026	1
Histidine metabolism	0.0786657	2	Insulin secretion	0.1130310	1
Phenylalanine metabolism	0.0845333	2	Regulation of lipolysis in adipocytes	0.1306146	1
beta-Alanine metabolism	0.0396028	2	Parathyroid hormone synthesis, secretion and action	0.0951003	1

Glycerolipid metabolism	0.0540798	2	Type II diabetes mellitus	0.0581703	1
Butanoate metabolism	0.0786657	2	Insulin resistance	0.1730973	1
Nicotinate and nicotinamide metabolism	0.1028906	2	Endocrine and other factor-regulated calcium reabsorption	0.0768157	1
Pantothenate and CoA biosynthesis	0.0351671	2	Amyotrophic lateral sclerosis	0.1306146	1
Nitrogen metabolism	0.0148078	2	Huntington disease	0.0581703	1
Aminoacyl-tRNA biosynthesis	0.0966528	2	Spinocerebellar ataxia	0.0675385	1
HIF-1 signaling pathway	0.0093237	2	Pathways of neurodegeneration - multiple diseases	0.2742870	1
Glutamatergic synapse	0.0025984	2	Cocaine addiction	0.0675385	1
GABAergic synapse	0.0033198	2	Amphetamine addiction	0.0860026	1
Taste transduction	0.0396028	2	Nicotine addiction	0.0675385	1
Mineral absorption	0.0330276	2	Alcoholism	0.0951003	1
Choline metabolism in cancer	0.0050084	2	Tuberculosis	0.0860026	1
Fatty acid degradation	0.3946779	1	Kaposi sarcoma-associated herpesvirus infection	0.0487102	1
Steroid biosynthesis	0.4417008	1	Pathways in cancer	0.2669531	1
Ubiquinone and other terpenoid-quinone biosynthesis	0.5106085	1	Renal cell carcinoma	0.0295108	1
Steroid hormone biosynthesis	0.6320106	1	Diabetic cardiomyopathy	0.3236549	1

P-value, obtained from the t-test of the substance in this group comparison; Number, the number of differential metabolites in this pathway.

Table S3. Analysis of the metabolic pathway of goat sperm in Y-27632 group and control group.

Pathway	Total	Hits	Raw p	Holm adjust	Impact
D-Glutamine and D-glutamate metabolism	5	3	0.000468	0.037905	1
Nitrogen metabolism	9	2	0.041713	1	0
Alanine, aspartate and glutamate metabolism	23	3	0.05148	1	0.4
Glycolysis or Gluconeogenesis	26	3	0.069899	1	0.09891
Glycine, serine and threonine metabolism	32	3	0.1142	1	0
beta-Alanine metabolism	17	2	0.13046	1	0.44444
Glycerolipid metabolism	18	2	0.14339	1	0.28098
Propanoate metabolism	20	2	0.16999	1	0
Butanoate metabolism	20	2	0.16999	1	0
Citrate cycle (TCA cycle)	20	2	0.16999	1	0.11316
Linoleic acid metabolism	5	1	0.17368	1	0
Pyruvate metabolism	22	2	0.19737	1	0.18754
Arginine and proline metabolism	44	3	0.22477	1	0.12721
Taurine and hypotaurine metabolism	7	1	0.23455	1	0.75
Cysteine and methionine metabolism	28	2	0.28174	1	0.10095
Valine, leucine and isoleucine biosynthesis	11	1	0.34337	1	0
Histidine metabolism	14	1	0.41489	1	0.07194
Pantothenate and CoA biosynthesis	15	1	0.43699	1	0
Pentose and glucuronate interconversions	15	1	0.43699	1	0.08333
Glyoxylate and dicarboxylate metabolism	16	1	0.45826	1	0
Fructose and mannose metabolism	19	1	0.51747	1	0.02273
Pentose phosphate pathway	19	1	0.51747	1	0.06757
Primary bile acid biosynthesis	46	2	0.52066	1	0.04033
Lysine degradation	20	1	0.53576	1	0.01493
Starch and sucrose metabolism	23	1	0.58664	1	0
Galactose metabolism	26	1	0.63204	1	0
Glutathione metabolism	26	1	0.63204	1	0.05534
Inositol phosphate metabolism	28	1	0.65955	1	0
Glycerophospholipid metabolism	29	1	0.67254	1	0.02442
Steroid biosynthesis	35	1	0.74086	1	0
Pyrimidine metabolism	37	1	0.76036	1	0
Fatty acid biosynthesis	38	1	0.76957	1	0
Fatty acid metabolism	39	1	0.77842	1	0
Biosynthesis of unsaturated fatty acids	42	1	0.80304	1	0

Aminoacyl-tRNA biosynthesis	64	1	0.91763	1	0
Steroid hormone biosynthesis	67	1	0.92694	1	0
Purine metabolism	68	1	0.92981	1	0

Total, the total number of compounds in the pathway; Hits, the actually matched number from the user uploaded data; Raw *p*, the original *P*-value calculated from the enrichment analysis; Holm adjust, *P*-value adjusted by Holm-Bonferroni method; Impact, the pathway impact value calculated from pathway topology analysis.

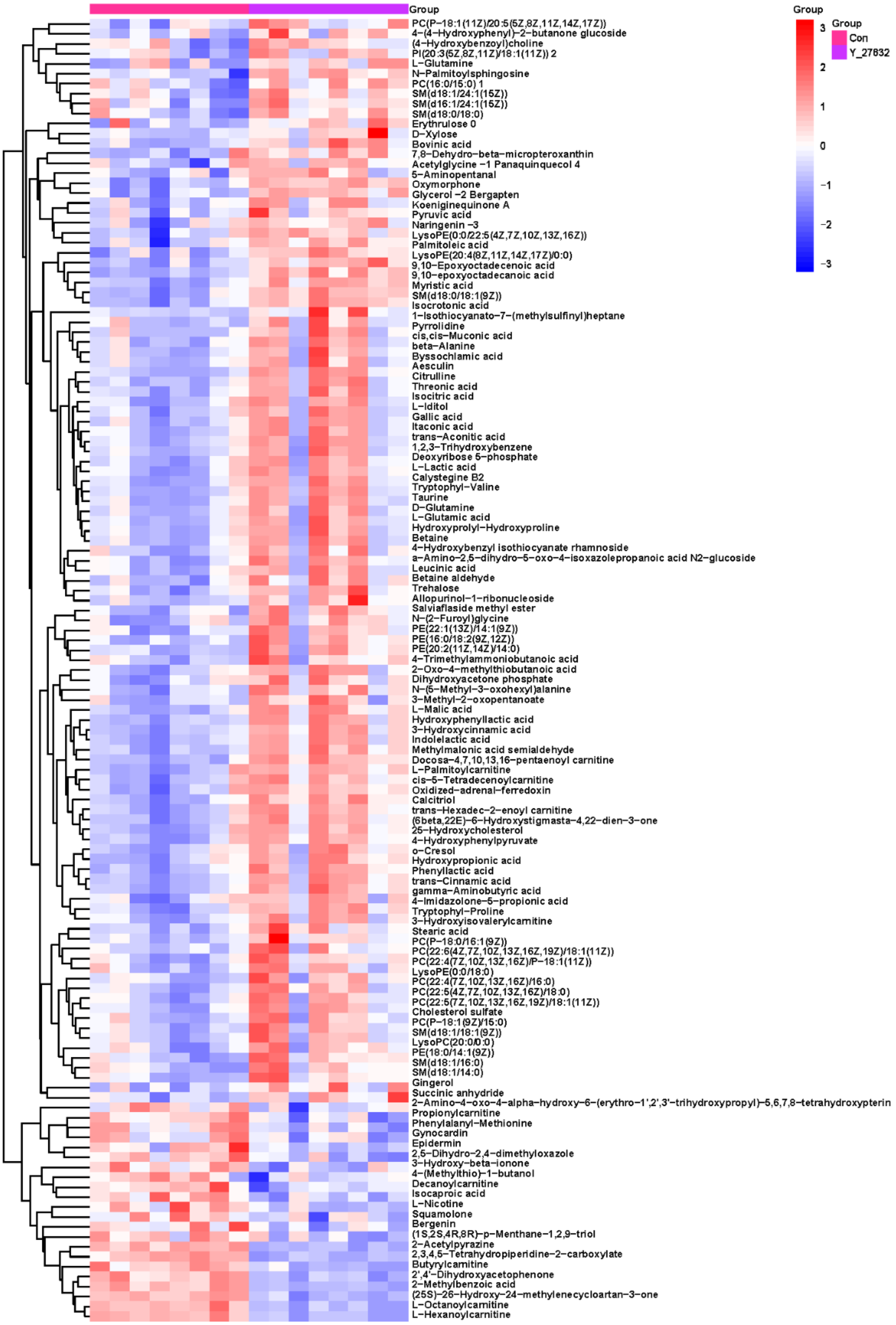

Figure S1. Hierarchical cluster analysis thermogram of differential metabolites of goat sperm in control group and Y-27632 group.

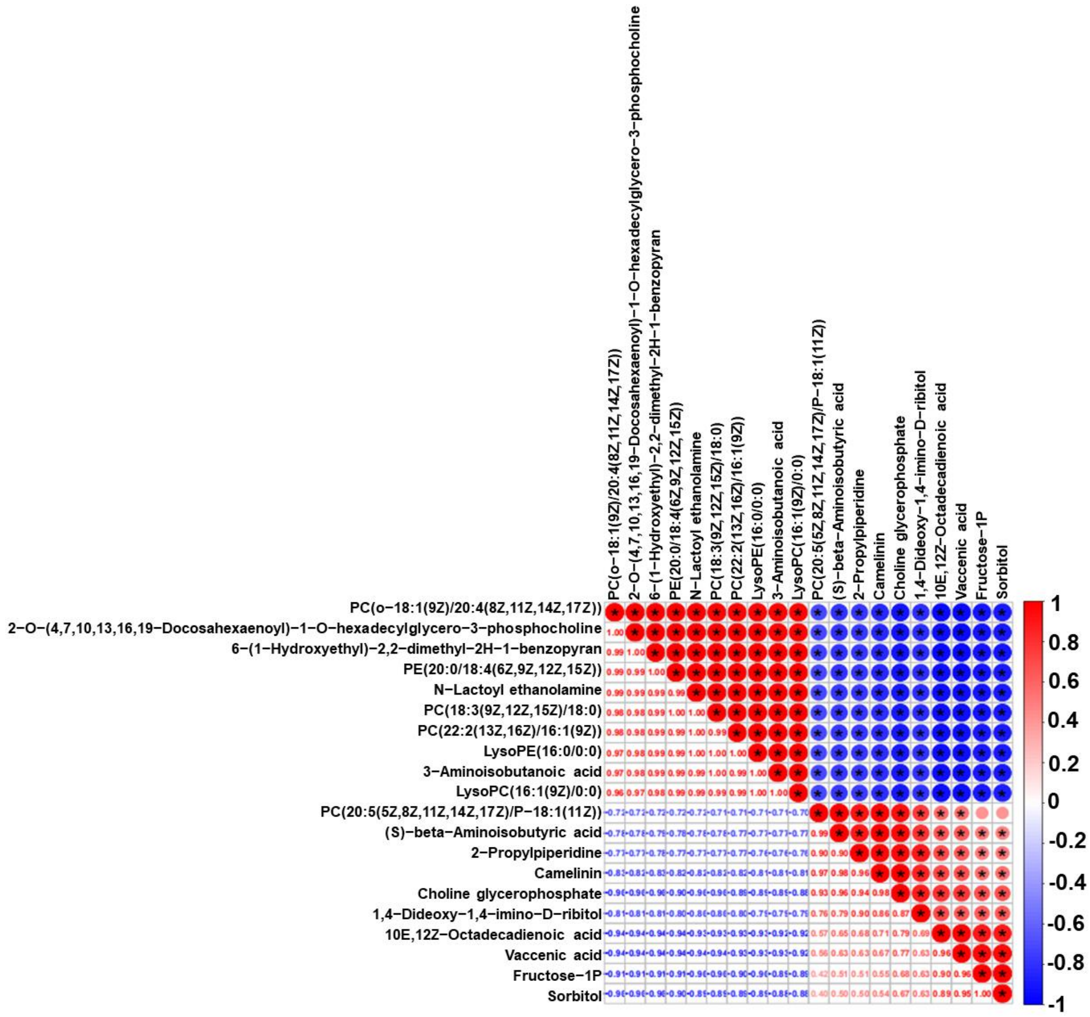

Figure S2. Hierarchical cluster analysis thermogram of differential metabolites of goat sperm and sheep sperm.
